# Forme fruste of isolated right ventricular endomyocardial fibrosis: a case report

**DOI:** 10.1186/1752-1947-8-94

**Published:** 2014-03-10

**Authors:** Matthew Bunte, Kenneth Liao, J Carlos Manivel, Emil Missov

**Affiliations:** 1 Division of Cardiology, University of Minnesota Medical Center, Minneapolis, MN 55455, USA; 2 Division of Cardiothoracic Surgery, University of Minnesota Medical Center, Minneapolis, MN 55455, USA; 3 Department of Laboratory Medicine and Pathology, University of Minnesota Medical Center, Minneapolis, MN 55455, USA

**Keywords:** Endomyocardial fibrosis, Venous thromboembolism, Transesophageal echocardiography, Cardiac magnetic resonance imaging

## Abstract

**Introduction:**

Endomyocardial fibrosis is a neglected tropical disease of unknown etiology and poor prognosis. It is endemic of tropical climates where it is the most common cause of restrictive cardiomyopathy in the second and fourth decades of life. A forme fruste of the disease is thought to be present in temperate climates where the diagnosis remains exceedingly rare.

**Case presentation:**

We describe a case of isolated right ventricular endomyocardial fibrosis in a 27-year-old Caucasian man from a temperate climate who presented initially with frank hemoptysis and pulmonary thromboembolic disease. We further describe the approach utilized in the diagnosis, the surgical treatment and the outcome of the disease.

**Conclusions:**

We conclude that endomyocardial fibrosis should be included in the differential diagnosis of apical cardiomyopathies in patients from temperate climates.

## Introduction

Endomyocardial fibrosis is the most common cause of restrictive cardiomyopathy in tropical climates [1]. It is characterized by idiopathic fibrosis of the endomyocardium and is frequently associated with thromboembolic disease, arrhythmias and complications of chronic heart failure [2,3]. A forme fruste of the disease is thought to be present in temperate climates where the diagnosis remains exceedingly rare [4].

## Case presentation

**Figure 1:**
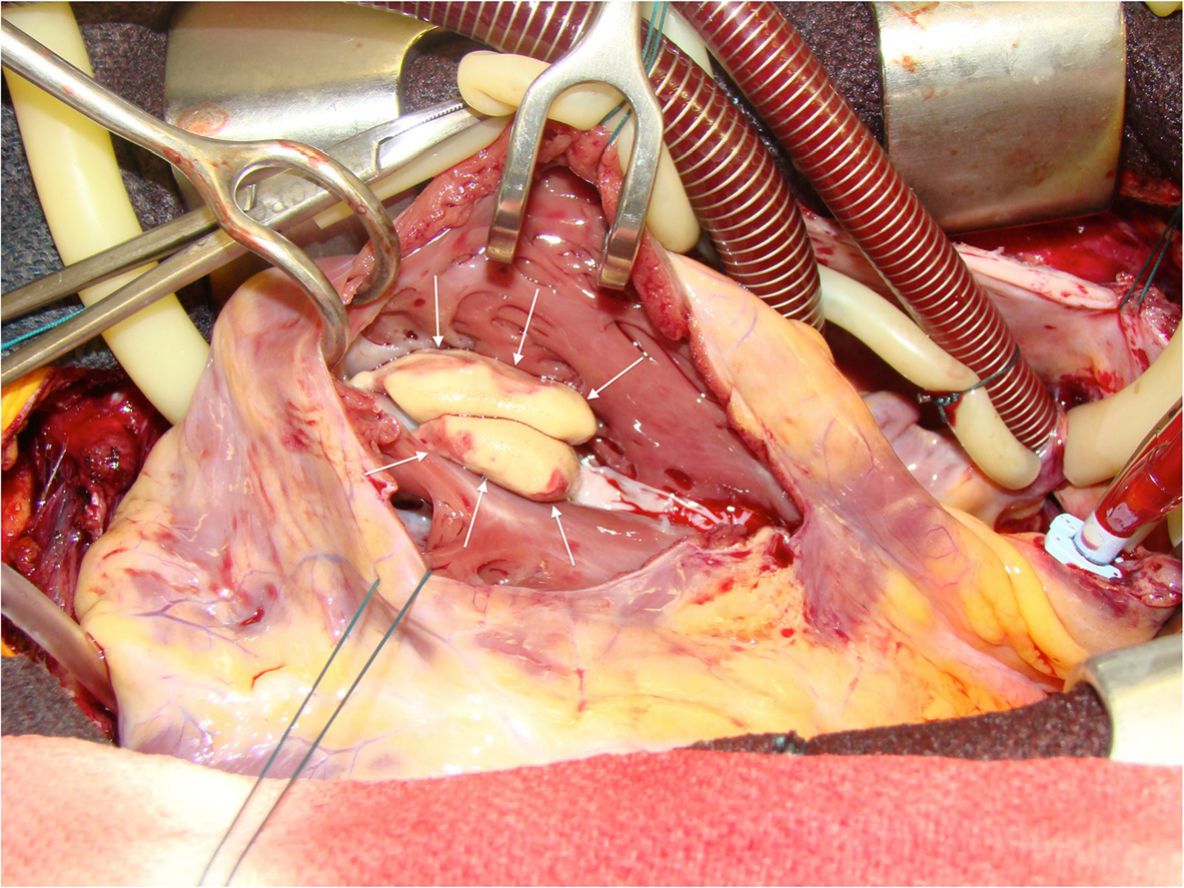
**Surgical findings.** A large, smooth, yellow mass (arrows) with lobulated heads was found in the right ventricular cavity following ventriculotomy.

**Figure 2:**
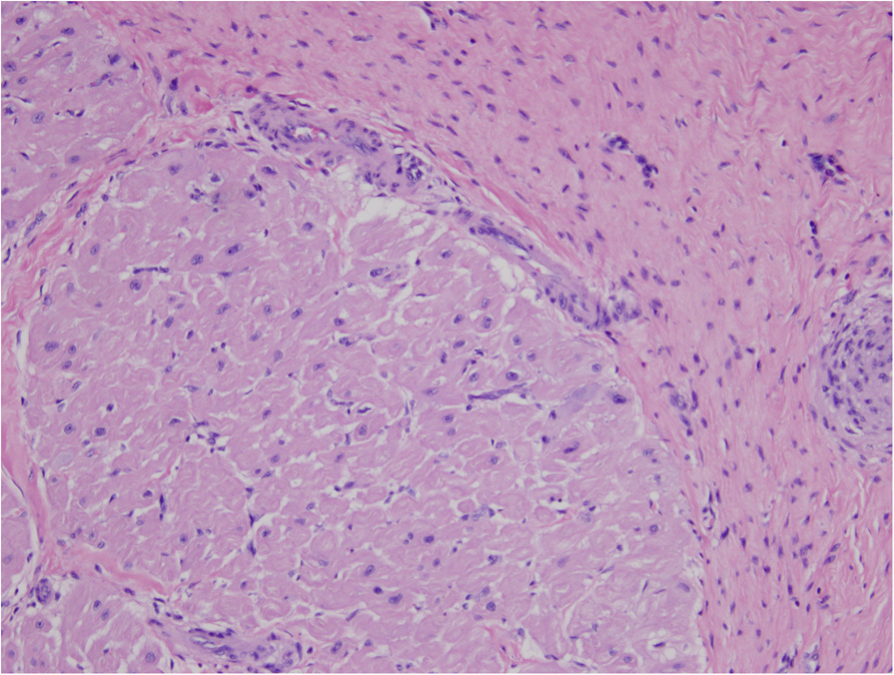
**Histopathological findings.** Markedly thickened fibrous endocardium (upper right) overlies normal myocardium (lower left). A layer of arterioles, venules and capillaries is seen at the interphase between the myocardium and thickened endocardium. Eosinophils are not present. (Hematoxylin-eosin stain; original magnification, ×400).

A 27-year-old Caucasian man presented with frank hemoptysis preceded by a two-month history of shortness of breath and non-productive cough. Prior to this subacute illness, he was healthy, had no travel history, and was a long-distance endurance athlete. An intravenously-contrasted computerized tomogram of the chest revealed bilateral pulmonary emboli and a filling defect within his right ventricle. Duplex lower extremity ultrasonography was negative for deep vein thrombosis. Transesophageal echocardiography revealed a mass at his right ventricular apex spanning proximally as pedunculated extensions through the tricuspid valve. Gadolinium-enhanced cardiac magnetic resonance imaging (MRI) confirmed a complex mass obliterating his right ventricular apex, insinuated between the chordae tendineae of the tricuspid valve and terminating in lobular stalks (Additional file 1). Surgical excision was recommended as the mass was felt to represent high risk for recurrent embolic events. The gross appearance of the mass was smooth, yellow and lobulated (Figure 1). Two distinct heads projected through the tricuspid valve and extensively insinuated between the chordae tendineae, requiring right ventricular endocardectomy with excision and replacement of the tricuspid valve. On histopathological examination, normal myocardium was covered by thickened fibrous endocardium with dense collagen, rare non-specific inflammatory cells with no eosinophils and a layer of arterioles, small veins and capillaries at the interphase with the myocardium (Figure 2). These findings are pathognomonic of endomyocardial fibrosis.

Anticoagulation therapy was initiated post-operatively. Extensive work-up for coagulopathy was negative. Peripheral blood and bone marrow biopsy were normal; in particular, the percentage of eosinophils was normal at 2%. Cytogenetic study of the bone marrow showed no evidence of a malignant process. Other causes of endomyocardial fibrosis, including parasitic infections, carcinoid tumor and systemic diseases, such as Churg Strauss syndrome, periarteritis nodosa and Behçet syndrome, were excluded.

## Discussion

The etiology and pathogenesis of endomyocardial fibrosis remain poorly understood. Several mechanisms have been postulated, including environmental and genetic factors, nutritional deficiencies, parasitic infections and eosinophilia. Recent studies have demonstrated antibodies against myocardial proteins and a high incidence of cardiotropic infective agents like cytomegalovirus, Epstein-Barr virus and *Toxoplasma gondii* in affected patients, suggesting a role for inflammation [5]. The prevalence of endomyocardial fibrosis peaks in the second and fourth decades of life with a male preponderance among all age groups. Approximately 55% of patients diagnosed with endomyocardial fibrosis have biventricular involvement and 17% have left-sided disease. The prevalence of isolated right ventricular endomyocardial fibrosis is 28% [1].

The diagnosis of endomyocardial fibrosis relies on echocardiographic criteria. The most salient features are a mass associated with one or both ventricles, typically the apices, and mural thrombus on the endocardial surface [3,4]. Cardiac MRI is a technique that provides additional information about ventricular hypertrophy, myocardial fibrosis, intracavitary masses and mural thrombosis [6]. Histopathological examination in endomyocardial fibrosis typically describes a thickened endocardium with elastic fibers and dense collagen, non-specific inflammatory cells, and a layer of arterioles, small veins and capillaries [5].

## Conclusions

Endomyocardial fibrosis is rare in temperate climates and should be included in the differential diagnosis of apical cardiomyopathies. Echocardiography and cardiac MRI each provide important information in the diagnosis of endomyocardial fibrosis; histopathological examination remains essential for the final diagnosis.

## Consent

Written informed consent was obtained from the patient for publication of this Case Report and any accompanying images. A copy of the written consent is available for review by the Editor-in-Chief of this journal.

## Abbreviations

MRI: Cardiac magnetic resonance imaging.

## Competing interests

The authors declare that they have no competing interests.

## Authors’ contributions

MB was a major contributor in writing the manuscript. KL obtained the surgical specimen and was a major contributor in writing the manuscript. JCM performed the histological examination of the surgical specimen. EM performed the transesophageal examination, image analysis and interpretation, and was a major contributor in writing the manuscript. All authors read and approved the final manuscript.

## Supplementary Material

Additional file 1 **Cine magnetic resonance imaging findings.** Cine cardiac magnetic resonance imaging study in the four-chamber view shows a large intracavitary space occupying mass in the right ventricle. The mass is polypoid and lobulated, and marginally mobile. Note the concordance with the surgical findings in Figure 1. Click here for file
